# A Review of the Clinical Progress of CT1812, a Novel Sigma-2 Receptor Antagonist for the Treatment of Alzheimer’s Disease

**DOI:** 10.3390/ph18050659

**Published:** 2025-04-30

**Authors:** Sara R. Steinfield, Daniel F. Stenn, Helen Chen, Bettina E. Kalisch

**Affiliations:** 1 Department of Biomedical Sciences, University of Guelph, Guelph, ON N1G 2W1, Canada; ssteinfi@uoguelph.ca (S.R.S.); stennd@uoguelph.ca (D.F.S.); hchen16@uoguelph.ca (H.C.); 2 Collaborative Specialization in Neuroscience Program, University of Guelph, Guelph, ON N1G 2W1, Canada

**Keywords:** Alzheimer’s disease, CT1812, clinical trials, ageing, sigma-2 receptor

## Abstract

Alzheimer’s disease (AD) is a neurodegenerative disease marked by the accumulation of toxic amyloid-beta (Aβ) oligomers. These oligomers are thought to cause synaptic dysfunction and contribute to neurodegeneration. CT1812 is a small-molecule sigma-2 receptor antagonist that is currently being investigated and tested as a potential disease-modifying treatment for AD. CT1812 acts by displacing Aβ oligomers into the cerebrospinal fluid and preventing their interaction with receptors on neurons. Preclinical studies and early clinical trials of CT1812 show promising results and provide evidence for its potential to slow AD progression. This review outlines the role of Aβ oligomers in AD, CT1812’s mechanism of action, and the effectiveness and limitations of CT1812 based on preclinical and clinical studies.

## 1. Introduction

Alzheimer’s disease (AD) is a multifactorial neurodegenerative disease which is characterized by progressive cognitive decline [1,2,3]. One of the pathological hallmarks of AD is the excess accumulation of the neurotoxic protein amyloid-beta (Aβ), which can form oligomers that bind to receptors on neurons and trigger neuroinflammation, oxidative stress, synaptic dysfunction, mitochondrial dysfunction, and neurodegeneration [1,4,5,6,7]. Current Health Canada-approved treatment options for AD, including acetylcholinesterase inhibitors and N-methyl-D-aspartate (NMDA) receptor antagonists, focus on managing symptoms rather than addressing the underlying pathology [1,8]. Consequently, ongoing research on drug development is investigating disease-modifying treatments that will slow AD progression [1,8,9].

CT1812, also known as Elayta, is a novel drug candidate that is being tested as a treatment for AD [10,11,12,13]. It is a small-molecule, brain-penetrant sigma-2 receptor antagonist which interferes with the binding of Aβ oligomers to neurons by displacing them into the cerebrospinal fluid (CSF) [10,11,12,13]. Preclinical studies and clinical trials have reported positive results regarding CT1812’s ability to target Aβ oligomers, making it a promising therapeutic candidate for slowing AD progression [10,11,12,13].

This review explores the therapeutic effects of CT1812, focusing on its mechanisms of action and the results from preclinical studies and clinical trials.

## 2. Background

### 2.1. Pathology of AD

Cognitive decline, a typical feature of AD, is attributed to synaptic dysfunction and neuronal cell death in the hippocampus and cerebral cortex, with cholinergic neurons being the most affected [4]. The hallmark pathologies of AD, thought to be responsible for this neuronal degeneration, are the accumulation of extracellular Aβ and intracellular neurofibrillary tangles (NFTs) made from hyperphosphorylated tau protein (p-tau) [4]. These pathologies contribute to neuronal inflammation, oxidative stress, impaired protein clearance, mitochondrial dysfunction, and, most importantly, the disruption of membrane protein trafficking and synaptic spine loss [5,7]. Altered protein trafficking and the loss of dendritic spines lead to malfunctions in long-term potentiation, resulting in memory and cognitive deficits [14,15]. The major cause of disruptions in protein trafficking and synaptic spine loss is the binding of soluble Aβ oligomers to the cell membrane [14,15]. Specifically, when Aβ oligomers bind to neuronal membranes, a decrease in the recruitment of membrane surface receptors occurs, leading to synaptic dysfunction which ultimately results in synaptic loss and the neuronal degeneration observed in individuals with AD [6]. However, if Aβ oligomers are removed before neuronal degeneration occurs, synaptic spines can often be regenerated, and cognitive function can be restored [7,10]. Since Aβ oligomer binding to cell surface receptors is a reversible and saturable process, pharmacological agents targeting this binding, through competitive or allosteric inhibition, could attenuate the Aβ-mediated disruptions in membrane trafficking and synaptic spine loss [6].

Recent research suggests that the sigma-2 receptor, which is expressed throughout the brain, plays a key role in mediating Aβ oligomer binding and significantly contributes to the membrane trafficking disruptions observed in AD [6,14]. The sigma-2 receptor is a heme-binding protein belonging to the membrane-associated progesterone receptor family [14]. These receptors can translocate from the endoplasmic reticulum to the plasma membrane in multiple cell types, including neurons, enabling their interaction with various cell membrane proteins [14,16]. These receptors have been shown to play a crucial role in maintaining the stability of surface receptors and interacting directly with proteins involved in regulating membrane trafficking [14]. Aβ oligomers bind to a specific oligomer receptor complex consisting of three main membrane proteins: LilRB2, the Nogo receptor, and cellular prion protein (PrPc) [14,15]. Importantly, the sigma-2 receptor is required for the stabilization and regulation of this oligomer receptor complex [10]. When Aβ oligomers bind to this oligomer receptor, the interaction disrupts normal receptor function and leads to changes in synapse-associated proteins, ultimately resulting in synaptic spine loss [10]. Highlighting the importance of the sigma-2 receptor in stabilizing the oligomer receptor complex in synaptic densities, a study conducted by Izzo et al. demonstrated that mice with a knock-down of the sigma-2 receptor had a 90% reduction in Aβ oligomer membrane binding [14]. Therefore, a drug that can competitively or allosterically inhibit Aβ oligomers from interacting with the sigma-2 receptor could act as a novel mechanism-based therapeutic for those with AD.

### 2.2. Mechanism of Action of CT1812

Cognition Therapeutics Inc. (Purchase, NY, USA) developed CT1812, a pharmacological agent designed to displace Aβ oligomers from sigma-2 receptors [10]. CT1812 is a small, lipophilic isoindoline sigma-2 receptor antagonist with high oral bioavailability and the ability to cross the blood–brain barrier (BBB) [10,11]. Notably, it is the first sigma-2 receptor antagonist to successfully progress to phase 2 clinical trials [10,11].

**Figure 1:**
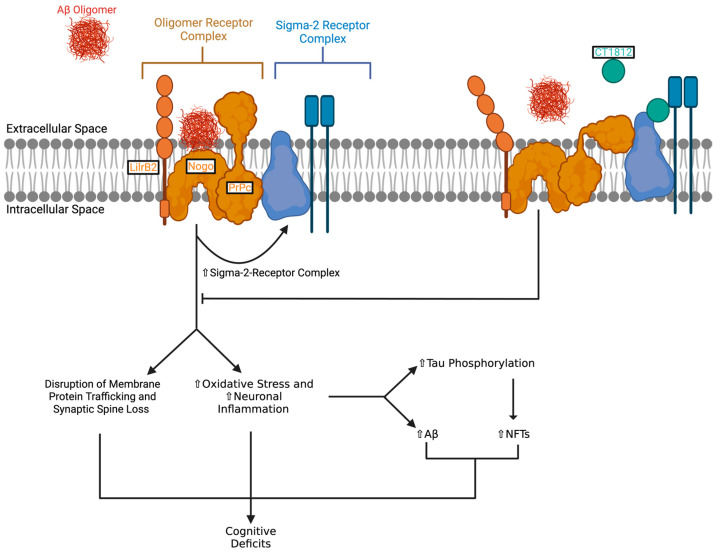
The mechanism of action of CT1812 in preventing Aβ oligomer-mediated cognitive deficits. On the left, the sigma-2 receptor complex stabilizes the oligomer receptor complex (LilrB2, Nogo, and PrPc), facilitating the binding of Aβ oligomers [10,14,15]. This interaction results in disrupted membrane protein trafficking, synaptic spine loss, increased oxidative stress and neuronal inflammation, elevated tau phosphorylation, and the accumulation of Aβ and NFTs, ultimately leading to cognitive deficits. On the right, CT1812 selectively binds to the sigma-2 receptor complex, inducing a conformational change that destabilizes the oligomer receptor complex, and releases bound Aβ oligomers, thereby preventing downstream pathological events and subsequent cognitive impairment [10]. Arrows indicate up regulation (↑), activation (→) and inhibition (⟙). Created using BioRender.com.

As seen in Figure 1, it has been hypothesized that CT1812 is a negative allosteric modulator of the sigma-2 receptor complex [10]. When Aβ oligomers are produced, they accumulate and bind to oligomer receptors, with the interaction stabilized by the sigma-2 receptor [10]. The binding of Aβ oligomers to this set of proteins alters its interactions with other proteins, and importantly, an upregulation of the sigma-2 receptor occurs, leading to a positive feedback cycle [10]. When CT1812 binds to the sigma-2 receptor complex with high affinity, it causes a conformational change in the sigma-2 receptor which destabilizes the Aβ oligomers [10]. The Aβ oligomers are then released and can be cleared from the brain through the CSF [10,11]. Destabilizing Aβ oligomers bound to neurons and facilitating their clearance from the brain presents a novel, mechanism-based approach that may enhance neuronal function and slow cognitive decline in individuals with mild-to-moderate impairment due to AD [10]. CT1812 has also been hypothesized to modulate tau phosphorylation by regulating tau kinases and/or phosphatases, though this mechanism has not yet been confirmed [10].

## 3. Current Research

CT1812 is the first sigma-2 receptor antagonist to reach the clinical trials stage [17]. Both preclinical studies and clinical trials have shown promising results, suggesting CT1812 could act as a novel therapeutic for those with AD.

### 3.1. Preclinical Studies

Initial studies investigated the effects of CT1812 analogues. In mixed hippocampal and cortical primary neuron cultures obtained from rats, these analogues reduced Aβ oligomer binding to neurons, attenuated membrane trafficking deficits, and reduced the loss of spines induced by Aβ oligomer treatment [6]. In a later study, CT1812 prevented synthetic Aβ oligomers from inducing membrane trafficking deficiencies by preventing oligomers from binding and displacing oligomers that were already bound to neurons [10]. Further, Aβ-mediated spine loss in these primary neuron cultures was reversed through the administration of CT1812, with spine improvement being dose-dependent but saturable [10]. These in vitro findings were supported by in vivo findings in animal models of AD. Drug analogues of CT1812 were given to mice treated with intrahippocampal Aβ to induce cognitive impairment and to mThy1-hAPP751 transgenic mice, which demonstrate excessive Aβ levels and progressive cognitive decline [6]. The results of this study demonstrated that for both AD models, when CT1812 analogues were administered at doses corresponding to 80% occupancy of the sigma-2 receptor, mice exhibited significant cognitive improvement compared to vehicle-treated mice [6]. A follow-up study reported similar improvements in spatial learning and memory in CT1812-treated transgenic mice, compared to those receiving the vehicle [10]. Overall, these results highlight the therapeutic potential of sigma-2 receptor antagonists like CT1812 and emphasize the need for further clinical studies in individuals with mild-to-moderate AD.

### 3.2. Clinical Trials

This section summarizes findings from human clinical trials investigating CT1812 and includes results from 10 completed clinical trials and data available from two ongoing trials. In these phase 1 and phase 2 trials, participants were recruited from different populations, including healthy subjects and AD patients, to assess various aspects of CT1812, such as its safety, pharmacokinetic profile, and possible involvement in disease-modifying pathways in AD. The results of the highlighted clinical trials are summarized in Table 1.

#### 3.2.1. Safety and Tolerability of CT1812 Across Clinical Trials

The published results from completed trials showed that CT1812 was well tolerated, with no major safety concerns, in both healthy individuals and AD patients across different age groups (Table 1) [10,17,18,19,20]. Adverse events (AEs) were minimal and generally mild to moderate, with the most common being headache [10,17,18,19,20]. The percentage of AEs was also similar across treatment and placebo groups, which suggests that the reported AEs might not directly result from CT1812 treatment [10,17,18,19,20]. The trials that have supported these conclusions include COG0101 (NCT02570997), COG0201 (SHINE—NCT03507790), COG0202 (SEQUEL—NCT04735536), and COG0102 (NCT02907567). The COG0101 (NCT02570997) trial specifically showed that CT1812 was safe across a wide range of doses and confirmed its ability to penetrate the BBB in humans, with >80% receptor occupancy (Table 1) [17]. This trial also demonstrated that CT1812 had no deleterious effects on cognitive function in the healthy elderly cohort (Table 1) [17]. Other trials, including COG0101 (NCT02570997), COG0108 (NCT05225389), COG0107 (NCT05248672), and COG0202 (SEQUEL—NCT04735536), provided information on the drug’s pharmacokinetic profile (absorption, metabolism, and excretion). One trial, COG0103 (NCT03716427), was unique as it assessed the interactions of CT1812 with four probe drugs (tolbutamide, midazolam, dextromethorphan, and omeprazole) to study its impact on the activity of certain cytochrome P450 (CYP) enzymes, which are hemoproteins involved in drug metabolism (Table 1) [13,21]. The results demonstrated that CT1812 has either no effect or a weak effect on the associated CYP enzymes and that clinically meaningful implications are unlikely (Table 1) [13,21]. This result provides insight into how CT1812 might interact with other medications used by AD patients.

#### 3.2.2. Amyloid Displacement and Tau Modulation

Three clinical trials examined CT1812’s role in amyloid displacement (Table 1). One trial, COG0102 (NCT02907567), demonstrated that AD patients in the CT1812 treatment group showed a significant increase in Aβ oligomers in CSF, which aligns with the drug’s mechanism of action and is consistent with preclinical data [10]. However, no significant differences between treatment groups were observed in Aβ40 and Aβ42 monomer levels [10]. The COG0104 (NCT03522129) trial also assessed Aβ monomer and oligomer displacement in CSF [22]. This trial included only three participants due to challenges in recruitment [22]. The AD patients who received CT1812 treatment in this trial showed an increase in CSF Aβ oligomers, with no change in Aβ40 and Aβ42 monomer levels, supporting the results from COG0102 (NCT02907567) [22]. The COG0105 (SPARC—NCT03493282) trial also showed no dose-related or treatment-related changes in CSF Aβ40 and Aβ42 monomer levels [19]. Similarly, the results from the COG0202 (SEQUEL—NCT04735536) trial found no differences in CSF Aβ40 and Aβ42 monomer levels between the treatment and placebo groups [20]. Lastly, the results from the interim analysis of CSF biomarker data in the COG0201 (SHINE—NCT03507790) trial differ from the previous trials [19]. In this analysis, CSF Aβ42 and Aβ40 monomer levels were significantly reduced by CT1812 treatment [18].

In terms of tau modulation, the COG0102 (NCT02907567) trial reported that AD patients in the CT1812 treatment group showed an overall reduction in p-tau, with multiple phosphorylation sites affected and no difference in unphosphorylated tau concentration detected, which suggests that CT1812 may have an impact on tau-related pathology in AD (Table 1) [10]. In contrast, the COG0105 (SPARC—NCT03493282) and COG0202 (SEQUEL—NCT04735536) trials reported no dose-related or treatment-related changes in CSF total tau and p-tau (Table 1) [19,20].

#### 3.2.3. Synaptic Function Modulation

In addition to safety, the potential for CT1812 treatment to preserve and improve synaptic function was investigated in some clinical trials (Table 1). One trial, COG0102 (NCT02907567), reported that in CT1812-treated AD patients, neurogranin (Nrgn) and synaptotagmin-1 (Syt1), which are proteins associated with synaptic damage, were decreased in CSF, suggesting that CT1812 may play a role in improving synaptic function and preservation [10]. In clinical trial, COG0201 (SHINE—NCT03507790), an interim analysis of CSF biomarker data did not provide insight into Nrgn and Syt1, but the trial identified pharmacodynamic biomarkers linked to pathway engagement (CT1812’s mechanism of action), disease modification biomarkers associated with favourable cognitive outcomes in the CT1812 treatment group, and disease modification markers which are altered in AD but normalized with CT1812 treatment [18]. Some of these biomarkers, such as neurexin 1 and 2, corroborate the results from both preclinical and clinical studies that show that CT1812 is protective of synapses [18]. The electroencephalogram (EEG) results in a pilot clinical study COG0202 (SEQUEL—NCT04735536) further support CT1812’s potential to preserve synaptic function, through its ability to improve established EEG markers of spontaneous brain activity in AD patients [20]. The same trial, however, also found no differences in CSF biomarkers (exploratory) like Nrgn [20]. Although the synaptic biomarker results from these trials seem promising, another trial, COG0105 (SPARC—NCT03493282), found no dose-related or treatment-related changes in CSF Nrgn or Syt1 after treatment [19]. Consequently, the mixed results regarding CT1812’s ability to preserve synaptic function indicate that further investigation is warranted.

#### 3.2.4. Cognitive Function

Not many clinical trials evaluated cognitive function as an outcome of CT1812 treatment, and the results were not conclusive or promising in the trials that did (Table 1). In the COG0102 (NCT02907567) trial, exploratory measures of cognitive function showed no significant difference between CT1812-treated and placebo groups, although no specifics were provided with respect to the cognitive tests used [10]. Similarly, the COG0105 (SPARC—NCT03493282) trial reported that CT1812 treatment did not significantly improve cognitive and clinical outcomes measured using the following clinical rating scales: Alzheimer’s Disease Assessment Scale—cognition subscale 11 (ADAS-Cog11); the Alzheimer’s Disease Cooperative Study—Activities of Daily Living (ADCS-ADL); the mini Mental State Exam (MMSE); and the Clinical Dementia Rating Scale Sum of Boxes (CDR-SB) [19]. In the interim analysis of the COG0201 trial (SHINE—NCT03507790), cognitive performance was measured using ADAS-Cog11 and a statistically non-significant but clinically meaningful 3-point improvement was observed in the CT1812 treatment group [18]. Lastly, the COG0202 (SEQUEL—NCT04735536) trial reported no significant differences between CT1812 and placebo for cognitive or functional outcomes measured using the Alzheimer’s Disease Cooperative Study—Clinical Global Impression of Change (ADCS-CGI-I); ADAS-Cog14; Amsterdam Instrumental Activities of Daily Living (IADL) Questionnaire; and Neuropsychological Test Battery (NTB) [21]. These results show that CT1812’s ability to displace Aβ oligomers into the CSF and its potential effects on synaptic function did not translate into improved cognitive outcomes, suggesting that a multifaceted approach to AD treatment may be necessary to ensure cognitive and clinical improvement in patients.

Overall, aside from CT1812’s safety profile, clinical trial results related to the drug’s ability to mitigate AD pathology are inconsistent and importantly, its potential to improve cognitive outcomes remains unclear (Table 1). More clinical trials are needed to establish CT1812’s efficacy, its involvement in mitigating AD pathology, and its effects on cognitive and clinical outcomes in AD patients.

### 3.3. Limitations and Future Directions

Although some clinical trials report positive results and support CT1812’s potential as a drug to slow down AD progression, several limitations must be addressed in future studies. A major limitation that many of the previously described human clinical trials faced was a small sample size [10,18,19,20,22]. As a result, these trials may lack the statistical power needed to detect differences between the treatment and placebo groups. This could also explain the inconsistency in reported results, as a difference in the measured outcome in the CT1812 treatment group may have existed; however, because of the small sample size, this difference was not detected. In addition, when the sample size is small and not sufficiently diverse, the findings of the trials may not be applicable to the broader AD patient population, limiting generalizability. AD patients may respond differently to different treatment options due to factors like age, sex, presence of comorbid illness, and other medications [23,24,25]. Thus, CT1812 may need to be combined with other therapeutic options to improve cognitive performance, which could explain why the trials that measured cognitive outcome did not report a significant difference between the placebo and treatment group. Another limitation is the variability between the study design and outcomes measured by the completed clinical trials, which makes it difficult to directly compare results and perform a meta-analysis to evaluate CT1812’s potential as a novel AD treatment [11,12,13,26]. Additionally, there is a lack of clinical trials that include cognitive assessments as a measured outcome, and as a result, CT1812’s clinical relevance remains unclear [11,12,13,26]. Lastly, different dosages of CT1812 were used in the clinical trials [11,12,13,26]. As a result, the optimal and most effective dose for CT1812 has not yet been determined, and factors, including efficacy, long-term safety, and tolerability, need to be considered, especially in elderly patients with AD.

Future studies should aim to address the above-mentioned limitations by conducting clinical trials with a larger sample size and a more diverse participant population to better evaluate CT1812’s safety and therapeutic potential. Additionally, these trials should incorporate cognitive assessments to provide more insight into CT1812’s ability to improve cognitive and clinical outcomes. Since AD pathology is multifactorial and as mentioned earlier, AD patients may respond differently to treatments, it would be beneficial for future studies to combine CT1812 with AD treatments that target a different aspect of AD pathology to assess whether cognitive and clinical outcomes are improved. To better evaluate the effectiveness of CT1812, it is imperative to identify biomarkers that can accurately monitor changes during treatment, provide insight into how the drug impacts AD progression, confirm its mechanism of action, and detect its therapeutic effects. These biomarkers would be crucial in determining the optimal timing and dose for treatment and allow for the identification of patients who respond to and will likely benefit from CT1812 treatment. Importantly, examining biomarkers will help reveal potential surrogate biomarkers that could be useful in future clinical trials and clinical settings. Long-term safety and efficacy studies (long treatment duration and extensive follow-up) evaluating multiple dosages would be necessary to determine the optimal dosage that ensures sufficient efficacy over an extended period while maintaining safety. Lastly, more studies are needed to replicate the findings of previous trials and validate the drug’s potential in AD treatment.

Overall, CT1812 shows potential and may be important in future AD treatment. However, further research is needed and must address the challenges and limitations outlined.

## 4. Materials and Methods

A literature search was conducted to determine the effects of CT1812 and its mechanism of action. The University of Guelph library database was used, with key words searched including “Alzheimer’s disease”, “treatment”, “CT1812”, “amyloid-beta”, “oligomers”, and “sigma-2 receptor”. Key scientific terms and references in relevant articles were used to find additional articles. Articles from English-language peer-reviewed journals related to preclinical studies and clinical trials on CT1812 were collected.

## 5. Conclusions

Both preclinical studies and clinical trials support the potential for CT1812’s use as a treatment to address excess Aβ accumulation in AD. The ability of CT1812 to displace Aβ oligomers from sigma-2 receptors and restore synaptic function makes it a promising candidate for therapeutic use in AD.

Although preclinical findings are promising, the results from clinical trials are inconsistent, with some showing positive results and others showing no difference from placebo groups. Limitations with clinical studies, such as long-term efficacy and safety, need to be overcome to fully validate CT1812’s potential as an AD treatment. Future research should focus on conducting large-scale, long-term clinical trials to determine optimal treatment time and dosage, to gain a better understanding of CT1812’s mechanism of action, identify clinically relevant pharmacodynamic biomarkers, establish long-term safety and efficacy profiles, and replicate previous findings to confirm the drug’s therapeutic potential.

## Figures and Tables

**Table 1:** Summary of CT1812 clinical trials highlighted in this paper, including study design, primary outcomes, and key findings.

Trial Name (ID)	Study Design	Primary Outcome(s)	Results
NCT02570997 (COG0101)	A two-part, phase 1, randomized, double-blind, placebo-controlled, parallel-assignment study in healthy adults evaluating safety, tolerability, and pharmacokinetics (PK) and determining the maximum tolerated dose (MTD) of CT1812. Part A (54 participants, aged 18–64) was a single ascending dose (SAD) study with 6 cohorts (10–1120 mg; 6 active–2 placebo) and one food-effect cohort (90 mg; n = 6). Part B (39 participants, aged 18–75) was a 14-day multiple ascending dose (MAD) study with 3 young adult cohorts (280–840 mg; 8 active–2 placebo) and 1 elderly cohort aged ≥65 (560 mg; 7 active:2 placebo). CSF sampling and cognitive testing were conducted in Part B.	Safety and Tolerability	**Safety and Tolerability** Safe and well tolerated Most AEs were mild or moderate AE rates comparable between CT1812 and placebo groups **Pharmacokinetics (PK)** Elderly subjects (≥65 yrs) showed ~1.5× higher exposure vs. younger adults CSF concentrations increased dose-dependently, confirming BBB penetration, with levels corresponding to >80% receptor occupancy **Cognitive Testing (Elderly Cohort)** No treatment-related cognitive decline was observed in elderly subjects receiving CT1812
NCT02907567 (COG0102)	A randomized, double-blind, placebo-controlled, parallel-group phase 1b/2a study evaluating the safety, tolerability, and PK of three once-daily doses of CT1812 (90 mg, 280 mg, and 560 mg) vs. placebo in adults with mild-to-moderate AD. A total of 19 participants (mean age 70.2) were randomized to receive either CT1812 or placebo for 28 days.	Safety and Tolerability	**Target Engagement** Dose-dependent increase in CSF CT1812 levels Significant rise in CSF Aβ oligomers vs. placebo, consistent with oligomer displacement No change in Aβ40 or Aβ42 monomers between groups or over time **Synaptic Effects (CT1812 vs. Placebo)** Reduced CSF Nrgn and Syt1 Proteomics revealed significantly altered synaptic proteins, including GSK3β and Wnt signalling components **Tau Phosphorylation (CT1812 vs. Placebo)** Reduced phosphorylation at multiple sites No change in unphosphorylated or total tau GSK3β levels trended lower, consistent with potential upstream modulation of tau kinases **Disease Modification (CT1812 vs. Placebo)** Partial normalization of AD-altered protein (involved in key AD-disrupted pathways)
NCT03716427 (COG0103)	A phase 1, single-centre, open-label, single-sequence drug–drug interaction study was conducted in 16 healthy adult volunteers (aged 18–55 years) to assess the effects of CT1812 on the PK of four CYP probe drugs (tolbutamide, midazolam, dextromethorphan, and omeprazole) administered before and after 6 consecutive daily oral doses of 560 mg CT1812 (steady state).	Area under the plasma-concentration time curve of CT1812 compared to the baseline values	Weak CYP2D6 (dextromethorphan) inhibition and CYP3A4 (midazolam) induction; no clinically relevant effects on CYP2C19 (omeprazole) or CYP2C9 (tolbutamide)
NCT03522129 (COG0104)	A multicenter, phase 1b, randomized, double-blind, placebo-controlled, parallel-group trial evaluating single-dose CT1812 (560 mg) vs. placebo in adults (50–80 years) with mild-to-moderate AD. Only 3 participants were enrolled due to recruitment challenges associated with invasive procedures.	Measuring the displacement of Aβ oligomers into CSF	**Safety** CT1812 was safe and well tolerated **Target Engagement** Significant exposure-dependent increases in CSF Aβ oligomer levels in CT1812-treated patients (vs. placebo), indicating oligomer displacement No significant change in Aβ40/Aβ42 monomers **PK/PD** Plasma and CSF CT1812 levels were measured and positively correlated with Aβ oligomer increases
NCT03493282 (SPARC; COG0105)	A phase 1/2 randomized, double-blind, placebo-controlled trial evaluating safety, PK, and PD of oral CT1812 (100/300 mg daily) in 23 mild-to-moderate AD patients (aged 50–85) over 24 weeks (+optional 24-week extension). Assessments included clinical measures, CSF biomarkers, and neuroimaging (SV2A PET, FDG-PET, volumetric MRI).	Safety and Tolerability	**Safety and Tolerability** CT1812 was safe and well tolerated Most AEs were mild, with headache being the most common **Neuroimaging (CT1812 vs. Placebo)** SV2A PET: No significant differences FDG-PET: No significant differences Volumetric MRI: Trend toward tissue preservation overall; nominally significant preservation observed in pericentral, prefrontal, and hippocampal cortices **Cognitive and Clinical Outcomes (CT1812 vs. Placebo)** No significant differences in ADAS-Cog11, MMSE, and CDR-SB ADCS-ADL: 300 mg group showed decline; pooled CT1812 groups (100 mg + 300 mg) showed a trend toward decline **CSF Biomarkers (CT1812 vs. Placebo)** No significant dose- or treatment-related changes in Aβ40, Aβ42, total tau, p-tau, Nrgn, or Syt
NCT03507790 (SHINE; COG0201)	An international, multicenter, randomized, double-blind, placebo-controlled phase 2 study in adults aged 50–85 years with mild-to-moderate AD. Approximately 144 participants were randomized 1:1:1 to receive oral CT1812 (100 mg or 300 mg) or placebo once daily for 6 months.	Safety and Tolerability	Results from an exploratory interim analysis (n = 24) **Safety** Safe and well tolerated; no serious AEs (SAEs) attributed to CT1812 **Cognitive Function (CT1812 vs. Placebo)** ADAS-Cog11: 3-point improvement in CT1812 group vs. placebo (clinically meaningful but not statistically significant) **CSF Biomarkers (CT1812 vs. Placebo)** CT1812 significantly reduced Aβ42 and Aβ40 Partial normalization of AD-associated protein dysregulation **Mechanistic Insights** Identified pharmacodynamic biomarkers altered by CT1812, confirming target engagement in synaptic, amyloid, and inflammatory pathways
NCT04735536 (SEQUEL; COG0202)	A phase 2, single-site, randomized, double-blind, placebo-controlled crossover trial evaluating CT1812 (300 mg) vs. placebo in 16 adults aged 50–85 with mild-to-moderate AD. The 29-day study comprised two 4-week treatment periods separated by a 2-week washout, with participants crossing over between CT1812 and placebo.	Safety and Tolerability Change in brain activity measured by global relative theta power via EEG assessments	**EEG Biomarkers (CT1812 vs. Placebo)** Global relative theta power: non-significant reduction; consistent trend in frontal, temporal, posterior, and central regions; significant decrease in the central region **Safety/Tolerability** Safe and well tolerated; only mild-to-moderate treatment-emergent AEs **Cognitive and Functional Assessments (CT1812 vs. Placebo)** No significant differences

## Data Availability

Not applicable. No new data were created or analyzed in this study.

## Abbreviations

The following abbreviations are used in this manuscript:

AD	Alzheimer’s disease
Aβ	Amyloid-beta
BBB	Blood–brain barrier
PrPc	Cellular prion protein
NMDA	N-methyl-D-aspartate
CSF	Cerebrospinal fluid
NFTs	Neurofibrillary tangles
p-tau	Hyperphosphorylated tau protein
AEs	Adverse Effects
PK	Pharmacokinetics
PD	Pharmacodynamics
CYP	Cytochrome P450
